# Robust Anti-tumor Response in a Patient with Metastatic Gastroesophageal Junction Adenocarcinoma on Long-term Maintenance Chemotherapy With Trastuzumab Alone: An Unusual Occurrence

**DOI:** 10.7759/cureus.11472

**Published:** 2020-11-13

**Authors:** Salman Haider, Hamad Ahmad, Saira Shah, FNU Neelma, Waqas Ullah

**Affiliations:** 1 Gastroenterology, Hayatabad Medical Complex Peshawar, Peshawar, PAK; 2 Internal Medicine, Hayatabad Medical Complex Peshawar, Peshawar, PAK; 3 General Surgery, Hayatabad Medical Complex Peshawar, Peshawar, PAK; 4 Internal Medicine, Abington Hospital Jefferson Health, Abington, USA

**Keywords:** adenocarcinoma, trastuzumab, gastroesophageal junction

## Abstract

Esophageal adenocarcinoma arises after a normal squamous mucosa undergoes metaplasia to the specialized columnar epithelium, which can then ultimately progress to dysplasia and subsequent malignancy. It typically presents at an advanced stage, and despite optimal management, the prognosis remains poor. Recently, the ToGA (Trastuzumab for Gastric Cancer) trial evaluated the role of trastuzumab (Herceptin®) in human epidermal growth factor receptor 2 (HER2) over-expressing gastric and gastroesophageal junction (GEJ) adenocarcinoma and showed improvement in overall survival when trastuzumab was added to the standard chemotherapy compared to standard chemotherapy without trastuzumab. However, data and experience are lacking in the literature regarding the use of trastuzumab outside of combination chemotherapy. The optimal length and safety of trastuzumab use as maintenance therapy in the long-term are also unclear in patients with gastric or GEJ adenocarcinomas after sustained remission.

We report a case of metastatic GEJ adenocarcinoma, where the patient first showed an exceptional response to standard chemotherapy combined with trastuzumab and subsequently could sustain a long-lasting remission on maintenance therapy with trastuzumab alone. Trastuzumab was ultimately discontinued owing to the lack of clear knowledge regarding its length and safety of use as maintenance therapy in the long-term.

## Introduction

Oesophageal adenocarcinoma is the eighth most common cancer and a leading cause of death in the world [1]. The two most common types of oesophageal cancers based on histology include squamous cell carcinoma and adenocarcinoma. Squamous cell carcinoma historically accounted for most cases of oesophageal cancer worldwide, but since the 1970s, the incidence of adenocarcinoma of the esophagus has been rising exponentially [2]. Oesophageal adenocarcinoma typically presents as a late-stage disease owing to its potential to metastasize early, and despite optimal management of the condition, the prognosis is typically grave. The five-year survival rate of the disease overall is usually less than 20% [3]. A multidisciplinary approach to the treatment of esophageal cancer is essential and requires input from experts in gastroenterology; surgical, radiation, and medical oncology; and often palliative care. Tumor location, staging, histologic type, medical comorbidities, and patient preference are factors that must be considered for selecting the proper treatment.

We present a unique case of metastatic oesophageal adenocarcinoma, where the patient unexpectedly sustained a complete remission for 35 months while being only on trastuzumab (Herceptin®) therapy. This case emphasizes the importance of therapies targeting the human epidermal growth factor receptor 2 (HER2) as they can dramatically change the otherwise poor prognosis of the disease to a more favorable one.

## Case presentation

**Figure 1:**
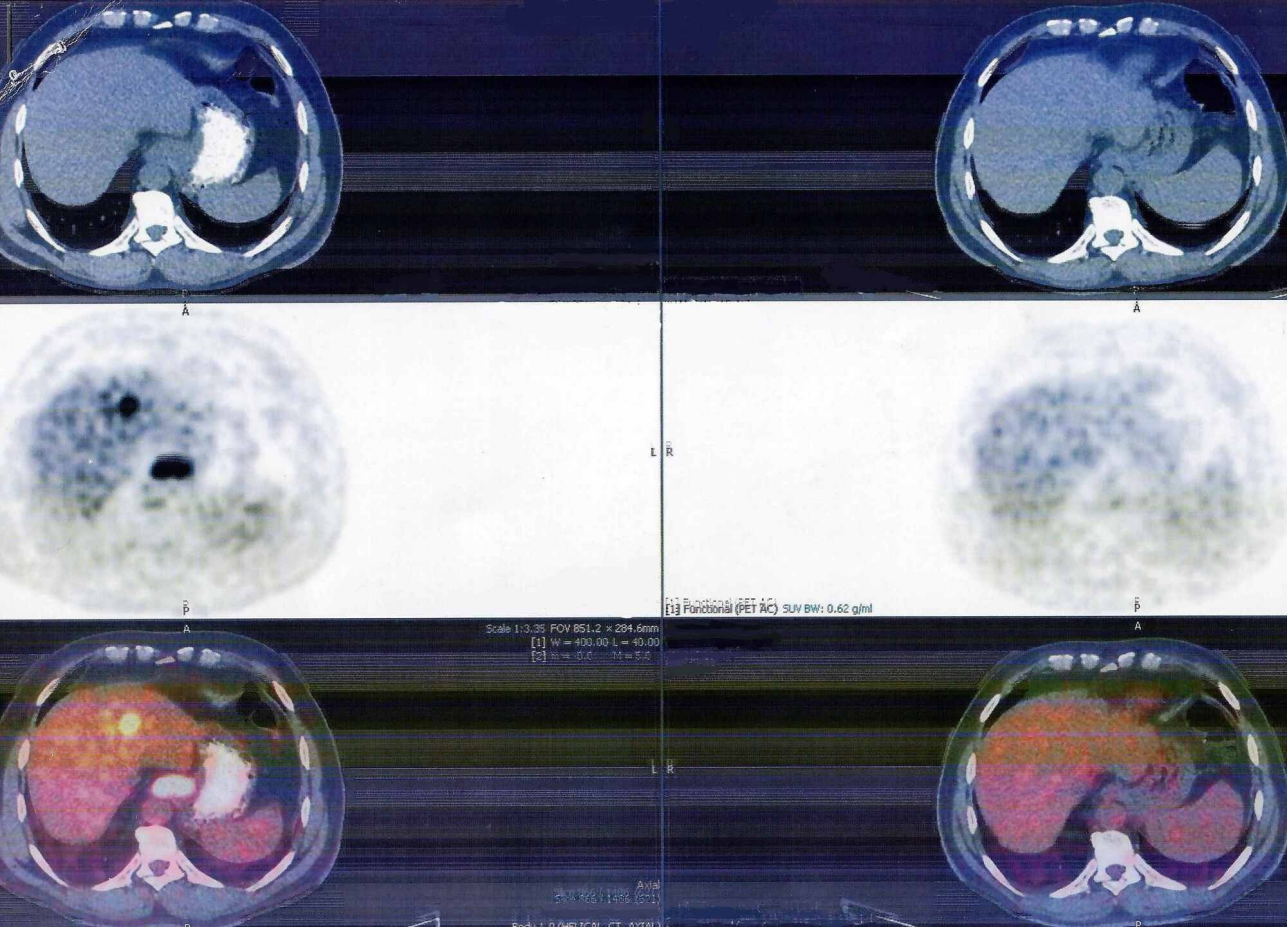
Positron Emission Tomography scan Towards the left side of the image is the initial scan at the time of diagnosis in August 2017, showing 18F-fluorodeoxyglucose avid lesions in the liver (metastatic lesions) and at the gastroesophageal junction. To the right is the last scan done in July 2020, which cannot show active metastatic disease process in response to trastuzumab monotherapy.

A 61-year-old man presented to the outpatient department with a three-week history of intermittent dysphagia for solids and a weight loss of 8 kg. He had a long history of gastroesophageal reflux disease for which he never sought care other than self-medication with proton pump inhibitors. A videofluoroscopic modified barium swallow study was done initially, which showed mild stasis of contrast at the gastroesophageal junction (GEJ). The patient subsequently underwent esophagogastroduodenoscopy (EGD) in August 2017, which identified a 3 cm ulcerated and fungating mass at the GEJ; biopsy of the mass confirmed a moderately differentiated (Grade 2) adenocarcinoma. Immunohistochemistry showed tumor cells overexpressing the HER2 receptors with a 3+ status, indicating a positive complete membrane staining in most tumor cells. Positron Emission Tomography (PET) scan and Endoscopic Ultrasound (EUS) staged the disease as T4, N2, M1. Multiple 18F-fluorodeoxyglucose avid hypermetabolic lesions in the liver and lungs were identified on PET scan, confirming a metastatic disease (Figure 1).

EUS revealed a circumferential hypoechoic mass with extension into the wall of the aorta (at 3 o’clock) and the right mainstem bronchus (at 6 to 8 o’clock), denoting a T4 disease which implies unresectability. Lower paraesophageal, common hepatic, and left gastric lymph nodes were positive for metastasis.

After a discussion with a multidisciplinary team, in September 2017 the patient was started on chemotherapy with palliative intent. The patient began taking a combination of chemotherapy comprising cisplatin and capecitabine plus trastuzumab. In January 2018, after six cycles of combination chemotherapy, a complete metabolic response was noted on restaging PET scan with normalization of metabolic activity of the previously active lesions. After achieving a complete response, cisplatin was discontinued while the patient continued taking capecitabine and trastuzumab for another 11 cycles until September 2018. A repeat PET scan in October 2018 did not show any disease activity, so capecitabine was also stopped, and the patient carried on with only trastuzumab as a maintenance therapy (consisting of 6 mg/kg of body mass every three weeks). Since then, multiple surveillance PET scans done every six months have consistently failed to reveal any proof of disease reappearance. Trastuzumab was ultimately discontinued in July 2020 after the PET scan showed repeated evidence of a complete metabolic response 35 months since the original diagnosis of advanced-stage disease (Figure 1).

Trastuzumab was well tolerated by the patient with no evidence of decreased cardiac ejection fraction on echocardiography. The performance status of the patient throughout the entire period was 0 (fully active and able to function without restrictions) as per the Eastern Cooperative Oncology Group criteria. All baseline investigations before and after initiation of chemotherapy were consistently unchanged except for mild thrombocytopenia (platelet counts 90,000 or above), which still persists despite discontinuation of all forms of chemotherapy.

## Discussion

Esophageal adenocarcinoma is a mortal disease. It typically presents at a later stage, and by the time symptoms appear, the disease has already progressed, and curative treatment is generally limited, and outcomes are poor. The goal of management is centered on alleviating symptoms, improving the quality of life, and extending life span.

A subset of patients (approximately 15-30%) with esophageal adenocarcinoma have amplification or overexpression of HER2, a human epidermal growth factor receptor that stimulates cell multiplication and resists apoptosis; hence strongly linked with a risk of recurrence and a grave prognosis [4]. This receptor was originally identified on breast cancer cells, but years later, gastric and esophageal adenocarcinomas were also found to overexpress it [4]. The discovery of HER2 also led to the development of drugs targeting it; trastuzumab, an anti-HER2 receptor monoclonal antibody, is one of such therapeutic drugs that, when added to the standard chemotherapy, has remarkably improved the otherwise worse outcomes of HER2 overexpressing cancers [4]. This was clear in the ToGA (Trastuzumab for Gastric Cancer) trial, which compared trastuzumab (administered at a dose of 6 mg/kg every three weeks) in combination with standard chemotherapy (cisplatin + either fluorouracil or capecitabine) versus standard chemotherapy alone for the treatment of HER2 overexpressing metastatic gastric or GEJ adenocarcinoma [5]. Subjects who received trastuzumab had a relatively higher response rate and improved overall survival compared to those who did not receive trastuzumab [5]. The results of the ToGA trial concluded that trastuzumab should be added to chemotherapy as a standard practice to manage HER2 overexpressing metastatic gastric or GEJ adenocarcinoma [5].

The dose and time-table of trastuzumab were analyzed further in the HELOISE trial, which determined that the standard-of-care for the management of HER2 overexpressing adenocarcinoma is standard-dose trastuzumab (a loading dose of 8 mg/kg followed by 6 mg/kg maintenance dose every three weeks) with chemotherapy [6].

In both trials mentioned above, trastuzumab was studied in combination with a standard chemotherapy regimen; based on our knowledge, very few studies have focused on the efficacy of trastuzumab as a maintenance therapy outside of combination regimens. Furthermore, the current literature is limited regarding the optimal length/duration of trastuzumab maintenance monotherapy in HER2-positive gastric or GEJ adenocarcinomas after prolonged remission has been achieved. In HER2 positive breast cancer, the length of maintenance therapy with trastuzumab is set at one year (52 weeks), as shown in the HERA (HERceptin Adjuvant) trial; research is warranted to evaluate whether this criterion may apply to HER2-positive gastroesophageal adenocarcinoma [7].

Our case report describes a rarely observed long-lasting remission for 35 consecutive months in a patient with metastatic GEJ adenocarcinoma on trastuzumab maintenance therapy alone, underscoring the need to study the efficacy of trastuzumab outside of combination regimens. The patient ultimately discontinued trastuzumab therapy owing to the lack of clear guidelines regarding the optimal length of trastuzumab maintenance monotherapy after sustained remission, mandating the need for future studies. Future research should also aim to evaluate the long-term safety of maintenance therapy with trastuzumab and the prognostic value of HER2 positivity in gastric or GEJ adenocarcinoma. 

## Conclusions

This case report describes a seldom seen, long-lasting remission in a patient with a primary diagnosis of metastatic GEJ adenocarcinoma to trastuzumab in combination with standard chemotherapy followed by a sustained remission on trastuzumab therapy alone; emphasizing the need to study the efficacy of trastuzumab in GEJ adenocarcinoma outside of combination regimens. Our case report also highlights the need for additional clinical trials necessary toward advancing our understanding of the safety and optimal duration of long-term maintenance therapy with trastuzumab in patients with gastric and GEJ adenocarcinoma after they have sustained remission. It also warrants the research to evaluate the prognosis and overall survivability of patients with HER2 overexpressing esophageal cancers as therapies targeting the HER2 receptor can alone remarkably change the prognosis of an otherwise deadly disease to a more favorable one.
